# Magnesiothermic conversion of the silica-mineralizing golden algae *Mallomonas caudata* and *Synura petersenii* to elemental silicon with high geometric precision

**DOI:** 10.3762/bjnano.5.65

**Published:** 2014-04-30

**Authors:** Janina Petrack, Steffen Jost, Jens Boenigk, Matthias Epple

**Affiliations:** 1 Inorganic Chemistry and Center for Nanointegration Duisburg-Essen (CeNIDE), University of Duisburg-Essen, Universitaetsstr. 5–7, 45141 Essen, Germany; 2 Department of Biodiversity, University of Duisburg-Essen, Universitaetsstr. 5–7, 45141 Essen, Germany

**Keywords:** biomineralization, golden algae, magnesiothermic conversion, silica, silicon

## Abstract

Chrysophyceae, also known as golden algae, contain characteristic, three-dimensional biomineralized silica structures. Their chemical composition and microscopic structure was studied. By high-temperature conversion of the skeleton of *Mallomonas caudata* and *Synura petersenii* into elementary silicon by magnesium vapour, nanostructured defined replicates were produced which were clearly seen after removal of the formed magnesium oxide with acid.

## Introduction

In nature, there are many biominerals which form silica-mineralized structures [1–3]. This process is also referred to as biosilification. The structural characteristics of these biominerals are species-specific [4–7]. For example, diatom frustules consist of silica which is assembled into complex 3-D structures and hierarchical forms [8–12]. These porous biominerals can serve as template for chemical conversion reactions, such as the calcium carbonate skeleton of sea urchins or the silica cases of diatoms [8,13]. In 2002, such a conversion reaction was first described in which biominerals were chemically transformed by a high-temperature conversion process [14].

Because it leads to delicate three-dimensional micro- or nanoscaled objects, the conversion of silica from biominerals into silicon is of potential interest for catalytic, biochemical, electronic and thermal applications. Furthermore, the use as electrodes in lithium batteries has been proposed [9,15–16]. Another advantage of such biofabrication approaches is the conversion of a large number of almost identical biological objects into defined materials.

Different approaches which start from porous silica-mineralizing organisms as template are possible. The deposition of gold or silver coatings on diatom frustules by thermal evaporation has been described [8]. Sandhage et al. have shown that diatom structures can be chemically converted at higher temperature, e.g., with magnesium to magnesium oxide or with calcium to calcium oxide. The principal reaction that occurs has been formulated as follows (s = solid; g = gaseous) [9,14]:


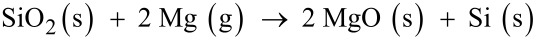


Sandhage et al. have found that silicon is formed first in a binary phase mixture of magnesium and silicon (magnesium silicide) and does not deposit on the cell walls, so that the deposition of magnesium oxide occurs onto the diatoms [9]. Shen et al. have demonstrated the chemical transformation of diatoms into silicon by magnesium [17].

The chrysophytes *sensu lato*, also commonly referred to as golden algae because of the yellowish colour of their chloroplast, belong to the Stramenopiles [18]. Chrysophytes, and stramenopiles in general are heterokont, i.e., they have a long flagellum bearing tripartite hairs and a short flagellum without such hairs. The flagella are used for locomotion [19]. The chrysophytes are usually photoautotrophic even though some lineages of the Chrysophyceae have secondarily reduced their plastid. The photoautotrophic chrysophytes form chlorophyll a and c and the accessory pigments α- and β-carotene. The characteristic golden-brown colour of the chrysophytes is based on the formation of the carotenoid zeaxanthin, a xanthophyll which masks the green colour of chlorophyll. They are mainly found in fresh water [20]. The chrysophytes *sensu lato* comprise the two classes Chrysophyceae and Synurophyceae. Some taxa, specifically the synurophycean genera *Mallomonas* and *Synura,* form silica scales.

Here we report on the analysis of the structure of the chrysophyte biomineral and their subsequent replication into elemental silicon with high geometric precision.

## Results and Discussion

**Figure 1:**
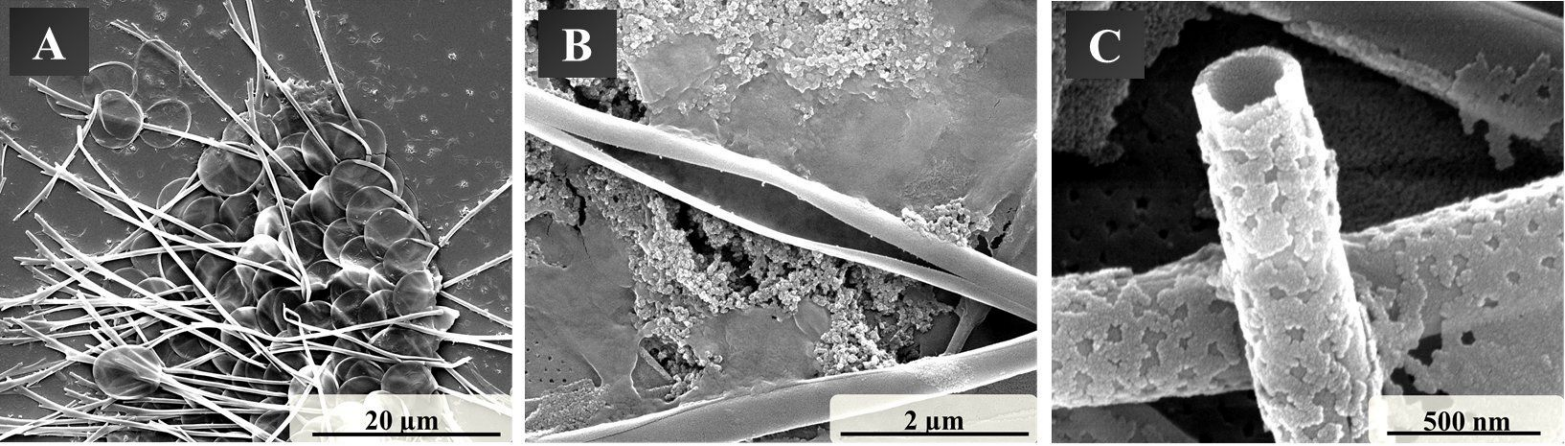
Scanning electron micrographs of the skeletal elements of *Mallomonas caudata* with an overview of spikes and shields (A), a broken spike (B), and a view into a broken spike (C).

Before the conversion experiments, the skeletal structure of the golden algae was investigated. Figure 1 shows scanning electron micrographs of the skeletal elements of *Mallomonas caudata*. These elements consist of a kind of "head" (the so-called shield) and a serrated spike. This spike is about 50 µm long, has a diameter of about 400 nm and a wall thickness of about 45 nm (Figure 1C). The spikes have an edge where they tend to break even by small mechanical stress (Figure 1B). The spherical shield consists of an outer ring and an apical pore field. The shield is about 8 µm in diameter.

**Figure 2:**
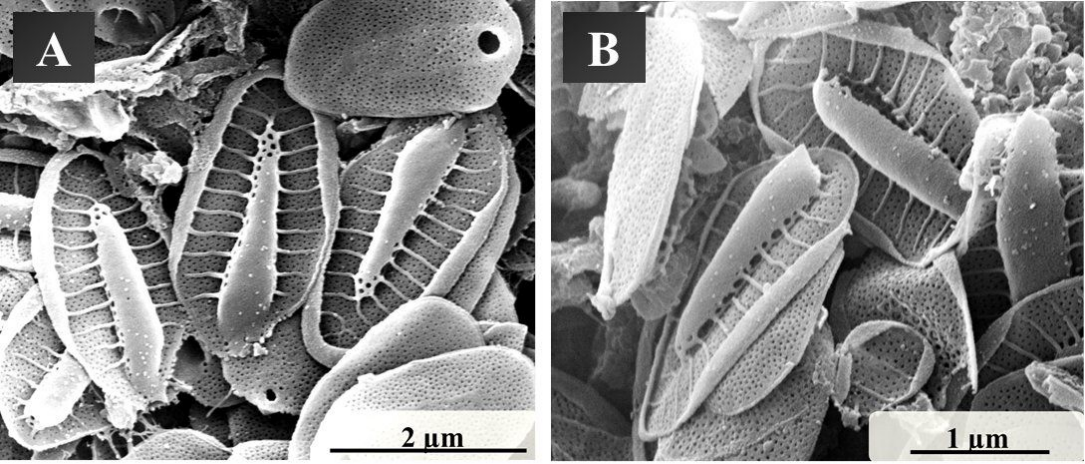
Scanning electron micrographs of the skeletal elements of *Synura petersenii*, showing the skeleton from various angles (A, B).

**Figure 3:**
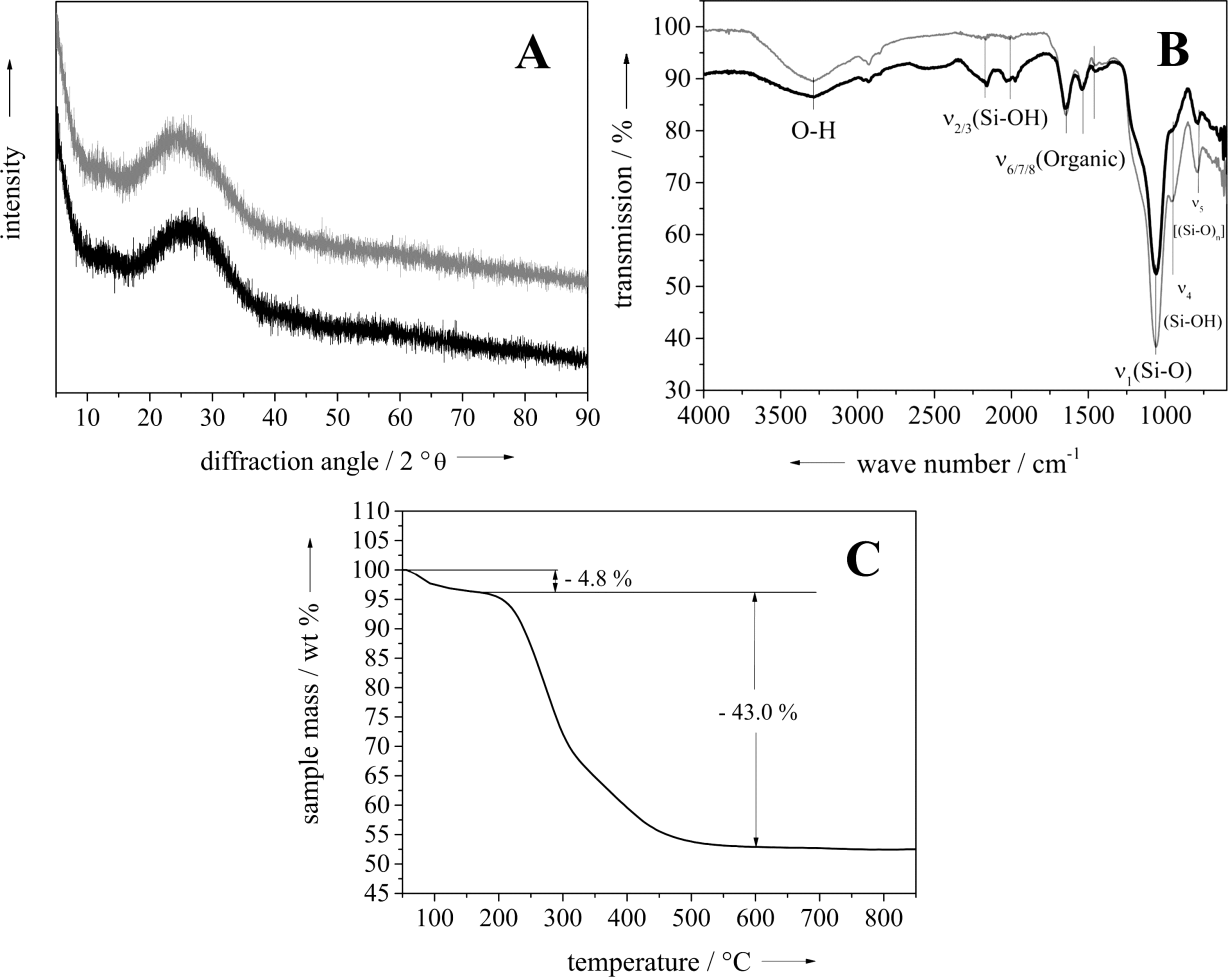
Characterization of the golden algae before the conversion. (A) shows powder diffractograms of *Synura petersenii* (black) and *Mallomonas caudata* (grey), (B) shows IR spectra of *Synura petersenii* (black) and *Mallomonas caudata* (grey), and (C) shows a thermogravimetric analysis of *Synura petersenii* in dynamic air atmosphere.

In Figure 2, scanning electron micrographs of *Synura petersenii* are shown. *Synura petersenii* consists of a mineralized skeleton with an average width of 1.5 µm and a length of 3 µm. They are composed of the base plate, the rims, the keel, the keel tips and the base plate hole. Similar to *Mallomonas caudata*, a pore field can also be seen in *Synura petersenii*. The golden algae were further characterized by X-ray diffraction, thermogravimetry and infrared spectroscopy (Figure 3).

*Synura petersenii* and *Mallomonas caudata* both contain only X-ray amorphous mineral, as expected for biogenic silica. IR spectra showed for both species the characteristic bands of water, silica and organic material. Thermogravimetry showed the decomposition in two steps. The first between 80 and 200 °C is associated with the loss of water (4.8 wt %). The second is attributed to the combustion of organic matter and also to dehydration of biogenic silica (43.0 wt % in total). The remaining 52.2 wt % of the sample can be assigned to inorganic silica, i.e., the dehydrated mineral ("SiO_2_").

**Figure 4:**
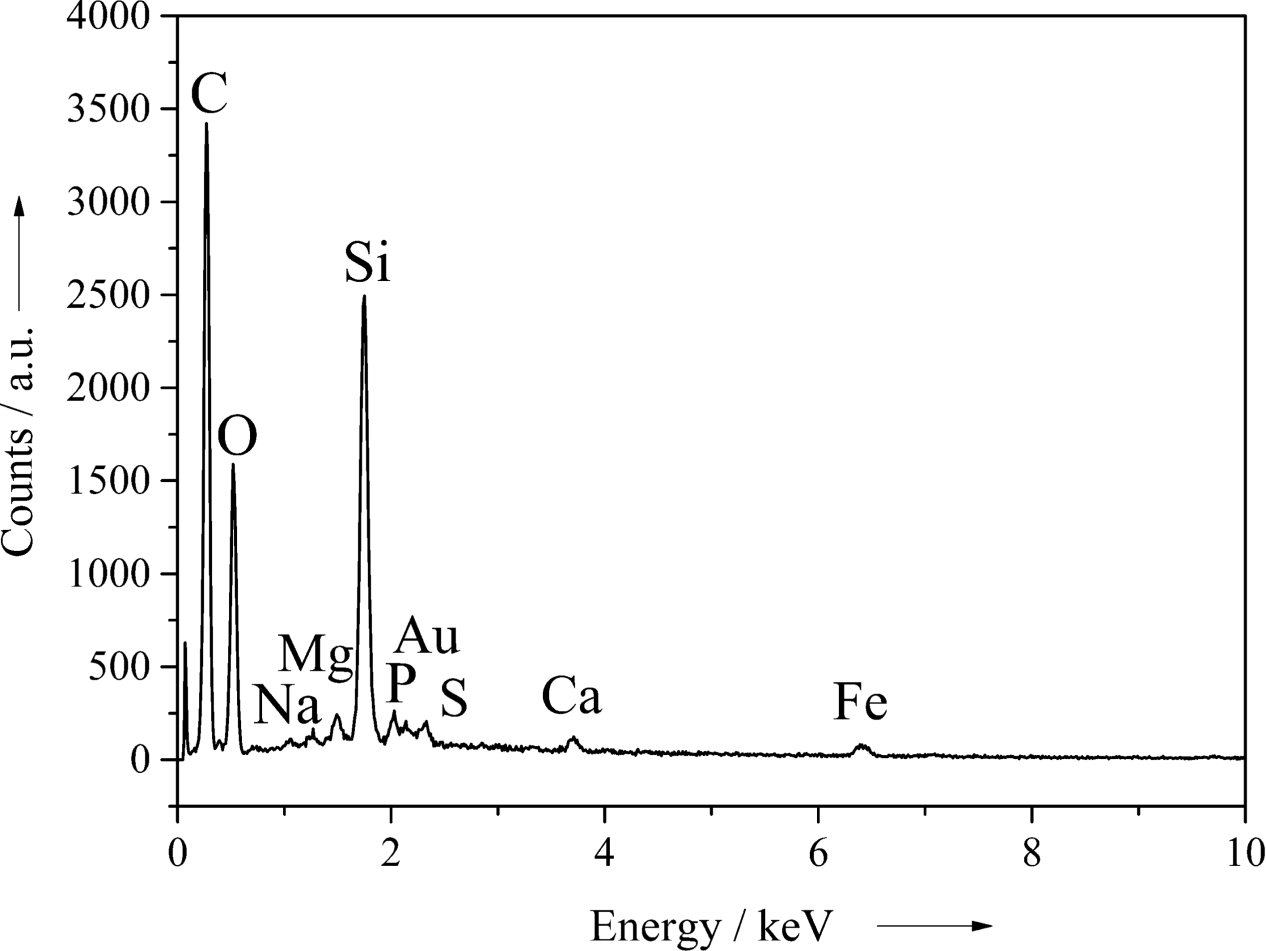
EDX analysis of golden algae (*Synura petersenii*) before the conversion.

The results of energy-dispersive X-ray spectroscopy (EDX) of the golden algae before the conversion are shown in Figure 4. Besides silicon, a couple of other elements were detected. The carbon and gold signals are due to the carbon sample holder and the gold sputtering, but the elements oxygen, sodium, magnesium, sulfur, calcium, and iron were present in the golden algae. However, it cannot be decided whether the metals were present in the organic matrix or as traces of salts from the culture medium.

**Figure 5:**
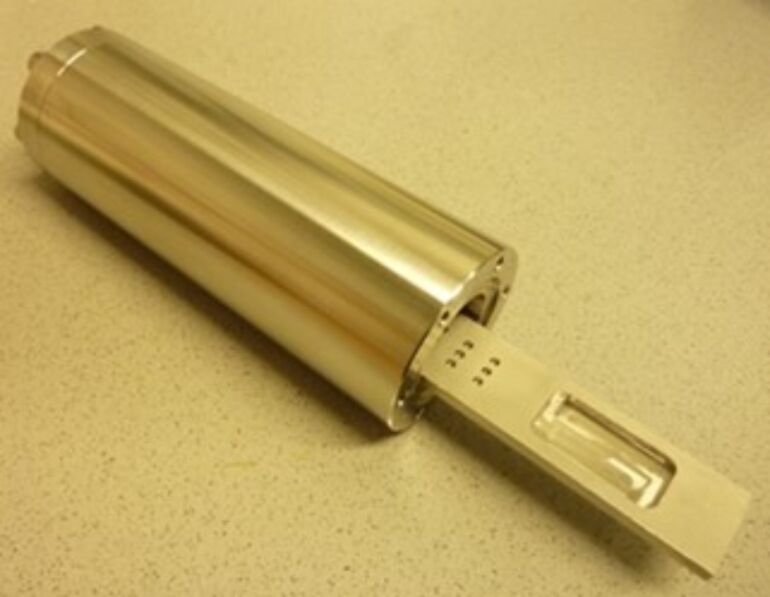
The tube reactor used for the chemical conversion of the golden algae.

The conversion of the skeletal elements of the golden algae *Synura petersenii* and *Mallomonas caudata* was carried out in a custom-made reactor consisting of heat-resistant steel (Figure 5). The reactor had an inner length of 18 cm and an outer length of 22 cm. The outer diameter was 7 cm; the inner diameter was 5 cm. Inside the tube was a rack with a large reservoir on both sides which each contained 3 g of magnesium powder (4 × 2 × 0.3 cm^3^). Golden algae were placed into 12 small wells, which were 0.2 cm in diameter and 0.3 cm in depth.

**Figure 6:**
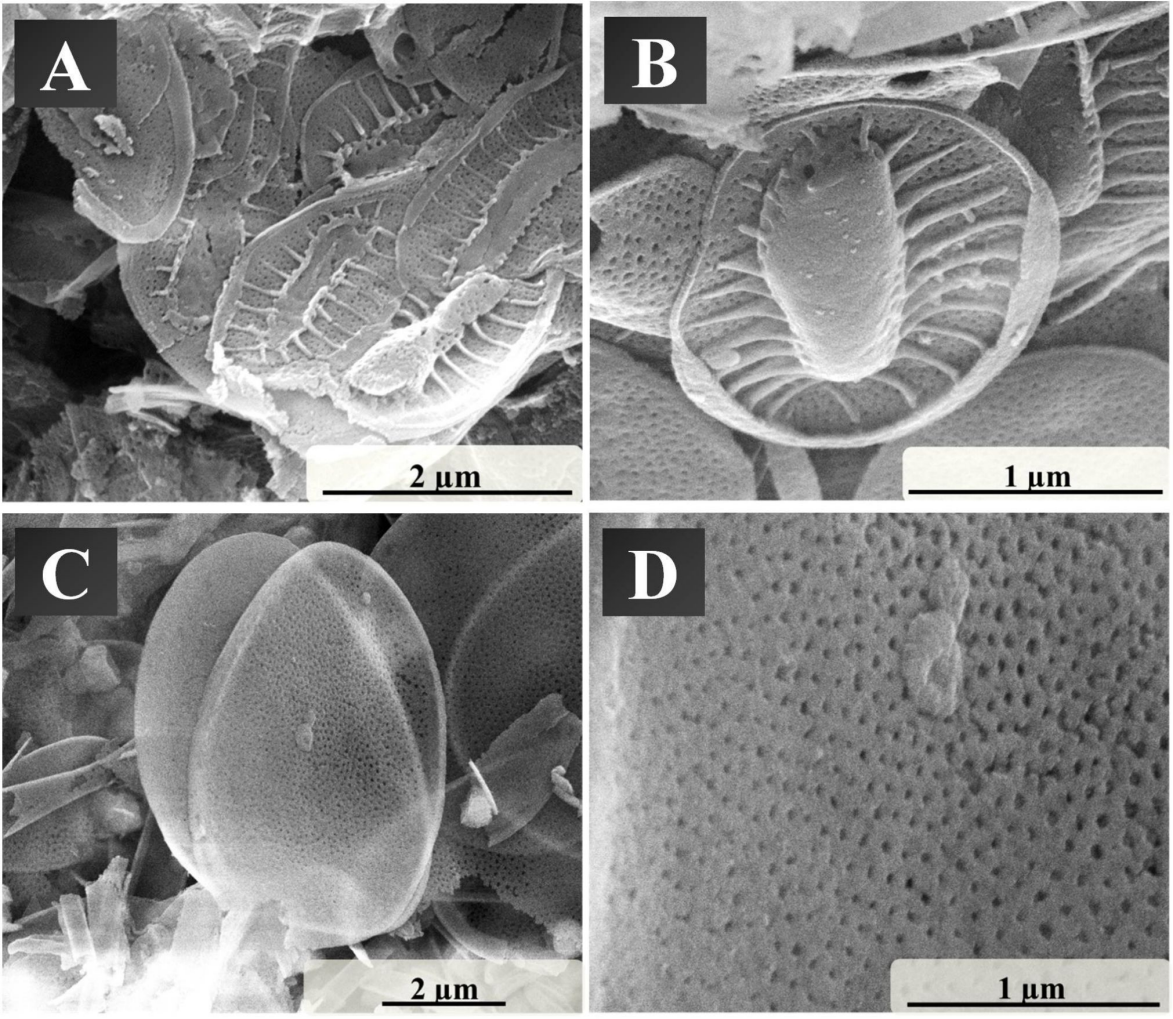
Scanning electron micrographs of the skeletons of *Synura petersenii* (A, B) and the shields of *Mallomonas caudata* (C, D) after conversion to silicon.

The dried golden algae were put into the wells (each filled about half) to carry out the conversion experiments. Then the reactor was tightly closed and transferred into a furnace, heated from room temperature to 900 °C within 4 h and kept at this temperature for 5 h. Finally, the furnace was freely cooled to room temperature and opened. The golden algae were removed gently from the wells. The formed magnesium oxide on the surface was carefully dissolved in 17% aqueous hydrochloric acid. The samples were dried at 37 °C in air. After the conversion to elemental silicon and removal of magnesium oxide, the structures of the golden algae were analysed by scanning electron microscopy (Figure 6).

The conversion led to a good replication of the initial biomineral structure of *Synura petersenii* (Figure 6A,B). All characteristic structural elements seen before conversion, such as the pore field or the wedge, were obtained. The "skeleton", as well as the keel and the base plate, interspersed with pores, were clearly visible. The fragility of the structures has been previously described [21]. The structures of *Mallomonas caudata* were more damaged by the chemical conversion and handling (Figure 6C,D). The spikes were mostly broken. The shield of the golden algae was preserved and shows a defined pore structure like before the conversion reaction. The size of the mineral structures was well preserved without noticeable shrinkage. Structures of 50 nm or less were well replicated.

**Figure 7:**
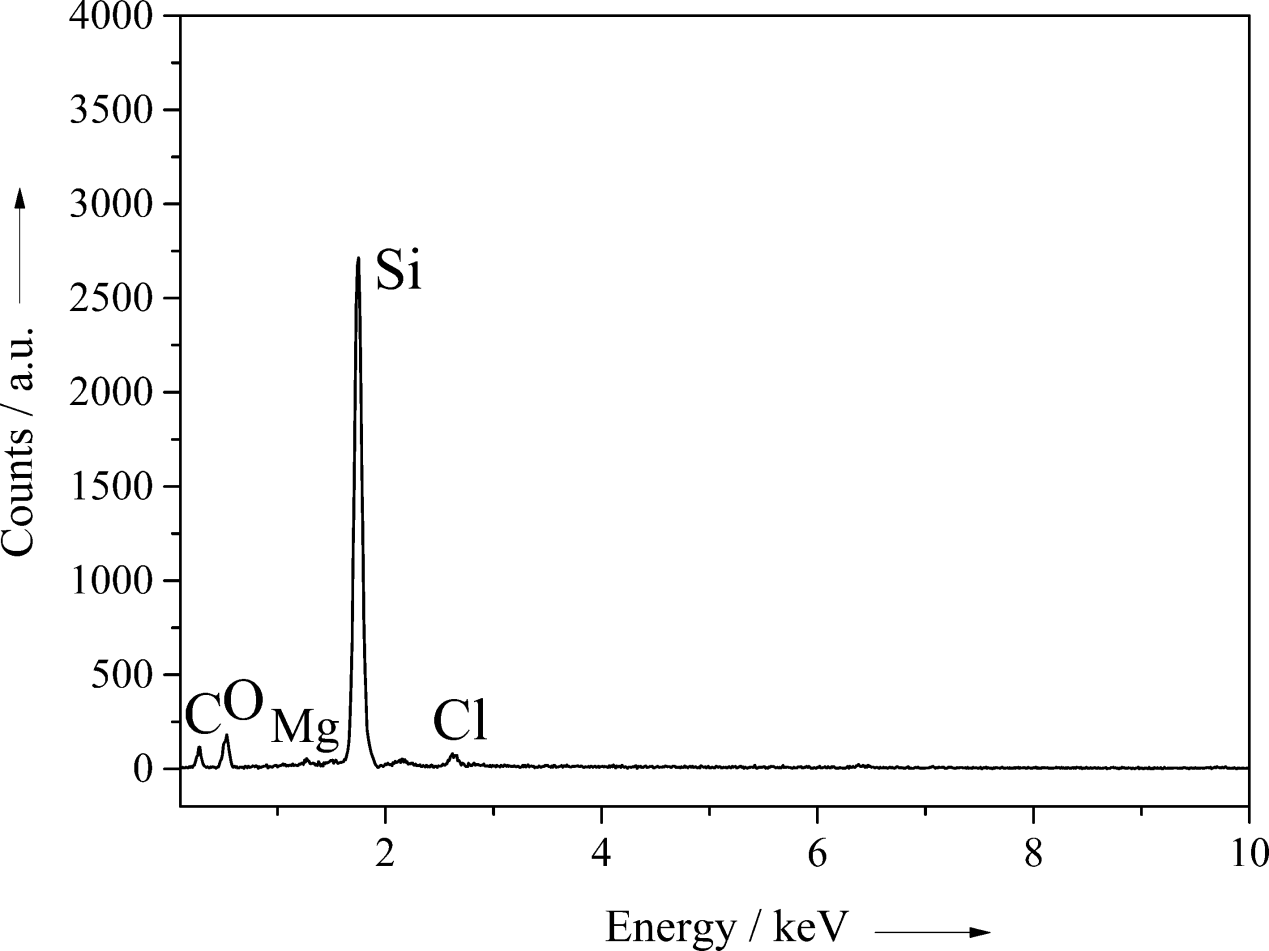
EDX spectra of *Synura petersenii* after conversion into silicon. The EDX spectra of *Mallomonas caudata* shows almost identical signals.

EDX-analysis showed the complete conversion of silica into silicon (Figure 7).

Besides silicon, only carbon (due to the sample holder), oxygen, and traces of magnesium and chlorine were detected. Similar results were reported by Bao et al. They reported that the conversion of diatoms with magnesium leads to silicon and magnesium oxide on the frustules. The latter was then washed out using hydrochloric acid and hydrofluoric acid (to remove traces of remaining SiO_2_) [22]. Sandhage et al. found that the conversion of diatoms with magnesium under flowing argon atmosphere does not lead to the formation of silicon because of the formation of a two-phase mixture of silicon and magnesium, which is not deposited on the surface of the diatoms. EDX spectra showed the absence of silicon and only the formation of magnesium oxide [9,14,23]. The formation of the mixed binary phase Mg_2_Si was also described in the literature, but the formation of the alloy phase occurs only as an intermediate that reacts in a subsequent step with silica to the products silicon and magnesium oxide [16].


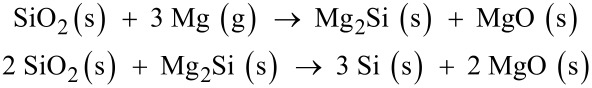


These equations correspond to our results because the formation of silicon was clearly detected by energy dispersive X-ray spectroscopy. Yamada et al. also showed that it is possible to convert magnesium silicide powder with magnesium into silicon [24]. In addition, magnesium silicide reacts with oxygen to magnesium oxide and silicon above 450 °C [25]. As our conversion is performed under an oxygen atmosphere, the presence of the binary mixed phase after the reaction can be excluded.

## Conclusion

The skeletal structures of the golden algae *Synura petersenii* and *Mallomonas caudata* were extensively characterized. They form typical delicate structures, such as the apical pore field, spikes and keels. The chemical conversion of the silica-mineralizing structure was conducted by magnesium powder in a tubular reactor at 900 °C. It was found that the delicate skeletal structures of the golden algae structures were well replicated. This shows that the magnesiothermic conversion route is well suited also for golden algae with their special skeletal features like rods and porous shells.

## Experimental

### Cultivation of *Mallomonas caudata* and *Synura petersenii*

We used the synurophytes *Synura petersenii*, strain WA18K-A, and *Mallomonas sp*., strain WA40K-F. Both strains were taken from the culture collection at the University of Duisburg-Essen (Department of Biodiversity). All strains were grown on modified WC medium at 15 °C and 90 µE [26]. In order to obtain a sufficiently high biomass for further analysis, the strains were serially transferred to higher culture volumes. Briefly, the cultures were first transferred to 100 mL Erlenmeyer flasks. After the strains reached the stationary phase (after approximately two weeks), the cultures were transferred to 500 mL Erlenmeyer flasks. The culture volume was then increased to 150 mL by adding fresh medium. After approximately ten more days, the culture volume was again doubled and after another ten days further increased to a final volume of 400 mL by adding fresh medium. After another ten days the biomass of 5 to 10 flasks was pooled prior to further analyses in order to obtain a sufficiently high yield of biomass. The golden algae were collected by filtration. The yield was dependent on the density of the biomass and varied strongly. Thus, the yield for *Synura petersenii* was between 2 and 5 mg per L culture medium. The yield for *Mallomonas caudata* was significantly lower with a maximum of 1 mg per L culture medium.

### Characterization

*Mallomonas caudata* and *Synura petersenii* were characterized by thermogravimetric analysis (STA 409 EP thermobalance from Netzsch; heating from 30 to 1200 °C with 2 K min^−1^ in dynamic air atmosphere), scanning electron microscopy (SEM, ESEM Quanta 400 FEG instrument with gold-palladium-sputtered samples), X-ray diffraction (XRD, Bruker D8 Advance powder diffractometer with Cu Kα-radiation), energy dispersive X-ray spectroscopy (EDX, ESEM Quanta 400 FEG instrument) and infrared spectroscopy (IR, Bruker Alpha-Platinum FTIR-Spectrometer).
